# Automated Feature Set Selection and Its Application to MCC Identification in Digital Mammograms for Breast Cancer Detection

**DOI:** 10.3390/s130404855

**Published:** 2013-04-11

**Authors:** Yi-Jhe Huang, Ding-Yuan Chan, Da-Chuan Cheng, Yung-Jen Ho, Po-Pang Tsai, Wu-Chung Shen, Rui-Fen Chen

**Affiliations:** 1 Department of Radiology, China Medical University Hospital, Taichung 404, Taiwan; E-Mails: yijhe@xuite.net (Y.-J.H.); 2062121@pchome.com.tw (P.-P.T.); wcshen@mail.cmu.edu.tw (W.-C.S.); t0271@mail.cmuh.org.tw (R.-F.C.); 2 Department of Electrical Engineering, National Chia-Yi University, Chiayi 600, Taiwan; E-Mail: dychan@mail.ncyu.edu.tw; 3 Department of Biomedical Imaging and Radiological Science, China Medical University, Taichung 404, Taiwan

**Keywords:** mammography, clustered microcalcification, texture features, support vector machines

## Abstract

We propose a fully automated algorithm that is able to select a discriminative feature set from a training database via sequential forward selection (SFS), sequential backward selection (SBS), and F-score methods. We applied this scheme to microcalcifications cluster (MCC) detection in digital mammograms for early breast cancer detection. The system was able to select features fully automatically, regardless of the input training mammograms used. We tested the proposed scheme using a database of 111 clinical mammograms containing 1,050 microcalcifications (MCs). The accuracy of the system was examined via a free response receiver operating characteristic (fROC) curve of the test dataset. The system performance for MC identifications was *Az* = 0.9897, the sensitivity was 92%, and 0.65 false positives (FPs) were generated per image for MCC detection.

## 1. Introduction

Cancer is one of the major causes of death in the World. There are many types of cancers, and its rates of occurrence differ, not only between men and women, but also between geographical areas. Breast cancer is the most common type of cancer among women in many developed countries [1]. Like most cancers, breast cancer has a high mortality rate, but is difficult to diagnose before the development of signs or symptoms. Early diagnosis and treatment plays an important role in improving survival rates and prognoses. For this reason, regular mammography and sonography screenings are recommended for women over forty years of age [2].

For the early detection of breast cancer, mammography is considered to be more effective than other imaging modalities at recognizing the typical sign of cancer—microcalcifications (MCs)—in the breast tissue. The appearance of MCs in a clustered distribution is considered to be a strong indicator of malignancy [3,4]. After a verification biopsy, there is a 15–34% chance of a malignant lesion developing in the MC tissue [5,6]. Clinically, clustered microcalcification is a meaningful indicator for breast cancer during mammography [7]. However, experts encounter a number of problems when manually reading mammograms: (1) dense breasts can cause low contrast between MCs and normal tissue, which results in reading difficulties, and (2) there exists a risk that radiologists might miss some subtle abnormalities. Moreover, analyzing a large number of mammograms generates a heavy workload for radiologists. Therefore, having a CADe system is helpful in clinical settings.

Computer-aided detection systems were developed to assist radiologists in reading mammograms many years ago. The first CADe system received FDA approval in 1998 [8]. Clinically, radiologists often use a CADe system to detect lesions and then make diagnoses. The system is considered to be a promising approach that may improve the sensitivity of interpreting mammograms [9–11]. In addition, CADe systems reduce radiologists' reading loads. CADe systems that are combined with mammography machines are expensive to upgrade [12], especially when the software is not optimized for use with certain imaging machines. For this reason, many researchers have proposed their own algorithms to detect MCCs, although most researchers and clinicians may have access to a CADe system. One of the recent streams of research involves using different imaging modalities (mammography and MRI) to increase the sensitivity of computer-aided diagnosis (CADx) [13].

Many previous studies have proposed different CADe systems for mammogram scanning. These systems can be grouped into two categories: supervised and unsupervised methods. Generally, supervised methods have some essential processes:

- Detect the breast area; reduce the intensity resolution;
- Use some pre-processing techniques such as enhancement or filtering to find suspected MCs;
- Extract features from training sample sub-images;
- Training a classifier to distinguish MCs from noise to find useful features.

The first item is a pre-processing step that is not only able to enhance images, but also reduce the computation time required for the following steps. This step is needed because both the spatial resolution and intensity resolution of mammograms is large. However, the breast area might occupy only one-third to one-fourth of the entire mammogram. Pre-processing can be achieved via some basic image processing techniques. Rather than analyzing the whole image, applying classifications only to MC candidates obtained from the pre-processing step can decrease computation complexity [14].The next three steps are also critical. Most state-of-art algorithms apply supervised methods [15,16] rather than unsupervised methods [17]. For this reason, we proposed a fully automated scheme for feature set selection from the training database and applied it to MCC detection.

The most common features used for MC detection can be roughly divided into two categories. One category is morphological features such as area, shape, compactness, *etc*. The other category is textural features [18,19]. The limitation of using morphological features depends on the image's spatial resolution [20] and the robustness of the MC segmentation algorithms [21]; the more precise the extraction of the MC shape, the better the classification performance. However, in some of our cases, the contrast between MCs and the surrounding tissues was very low, and it was difficult to segment MCs clearly, especially in younger women who have more dense breasts. For this reason, the morphological features proposed in [21] were inappropriate for MC detection in our test cases. Instead, the shape information was used during the knowledge-based classification as a noise reduction procedure in our study. In contrast, textural feature analysis seemed to be able to alleviate the MC segmentation problem, likely because it can capture textural changes in the MCs' surroundings.

The significance of this study is that we proposed a scheme that is able to automatically select discriminate features via SFS, SBS, and F-score methods. This scheme was applied to MC identification and MCC detection in digital mammograms. The structure has four stages, briefly described as follows. In the first stage, the mammogram was filtered so that only suspicious MC candidates remained. To do so, a hybrid filter consisting of a wavelet filter, a top-hat filter, and 15 Laws filters was applied to alleviate the problem of low contrast between MCs and surrounding breast tissue. This filtering not only lessened the low contrast problem but also reduced the tremendous computation time because only high-frequency components remained for further processing. In the second stage, all candidates were examined by a knowledge-based classifier to reduce the number of false positives (FPs). Furthermore, the remaining candidates were classified by support vector machines (SVM) via a set of features after an automatic feature selection, in which the optimal parameter sets for SVM were also determined. Finally, we clustered individual MCs to MCCs and marked the identified MCCs on the images as a result.

The rest of this paper is organized as follows: Section 2 introduces our mammogram database and methods of pre-processing, filtering, feature extraction, automatic feature selection, training, and classification. Our experimental results are shown in Section 3. We then discuss our method and methods from other groups in Section 4. Finally, the conclusions are provided in Section 5.

## 2. Methods

### 2.1. Datasets and Ground Truth

Fifty-two patients (cases) with clinical reports were collected, from which a total of 111 digital mammograms were acquired. The image gray-level resolution was 14-bit per pixel. Each patient had at least one craniocaudal (CC) view and one mediolateral oblique (MLO) view. All the mammograms, which were representative images containing MCCs, were acquired from China Medical University Hospital. The patient mammograms were selected by two radiologists, who selected mammograms that they both agreed contained precisely recognizable MCs. Patients whose mammograms were not able to be identified consistently by these two radiologists were excluded. To establish ground truth, all mammogram readings were performed by these two experienced radiologists independently. One radiologist was a senior clinician who has worked in this area for over ten years. The other radiologist was young and has worked more than two years. In each mammogram, a rectangle (or some rectangles) was drawn to enclose the MCCs, and a point was manually marked in the center of each MC. The rectangles were drawn as small as possible to cover the MCCs. The manually identified MCs were set as the gold standard used as the ground truth to which the automated results were compared.

**Figure 1.**
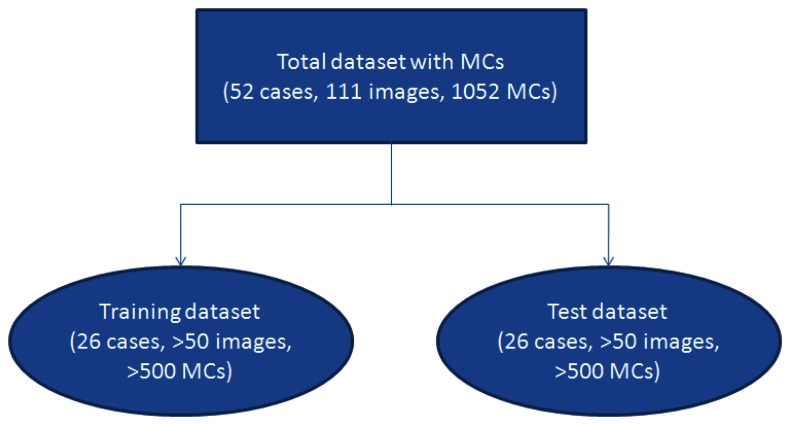
Two-fold cross-validation test.

To make a statistical analysis, we used 2-fold cross-validation [22,23] to test our algorithm. The dataset was randomly separated into two subsets. One subset containing 26 cases (more than 50 images) was used as the training dataset, and the remaining 26 cases (more than 50 images) were grouped into the test subset. The validation scheme is shown in Figure 1. We used 2-fold cross-validation instead of other methods such as 10-fold cross-validation because it could estimate the system performance more reliably.

### 2.2. Image Pre-Processing, Filtering, and Feature Extraction

**Figure 2.**
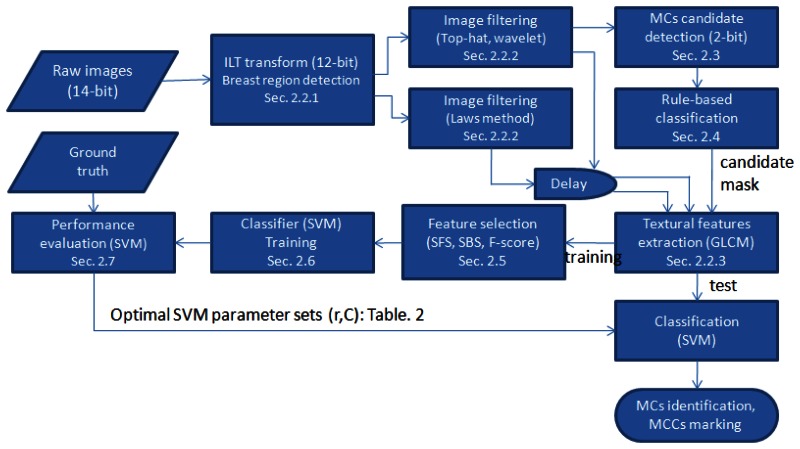
A flowchart of the proposed method.

Figure 2 shows a flowchart of the proposed method. The details of each procedure are discussed in the following subsections.

#### 2.2.1. Inverted Logarithmic Transform (ILT) and Breast Region Detection

To avoid the impact of manufacturers' proprietary pre-processing steps, we used raw images as the sources. In the clinic, mammograms are usually viewed as an inverse of the raw image. The raw images were transformed from 14 to 12 bits, and then the images were negatively transformed to fit clinical reading conditions by applying an inverted logarithmic transformation (ILT) [24]. This step not only reduced the intensity resolution but also enhanced the image [25]. The algorithm was performed on the 12-bit images after the ILT transform. The details of ILT were presented in [24].

After image transformation, the breast region was determined to speed up the later computations. To do so, a binarization procedure was applied. The threshold value was automatically determined using Otsu's method [26], which minimizes the within-class variance. Next, region growing [25] was performed. The largest region was considered to be the breast region, and the remaining regions were ignored to reduce the computation load.

#### 2.2.2. Image Filtrations: Top-Hat, Wavelet, and Laws

Two popular filtrations were utilized. Top-hat filtration was used to alleviate the uneven background problem, whereas wavelet filtration was used to obtain high-frequency components (*i.e.*, MCs).

##### Top-hat

Top-hat filtering was used to segment spot-like objects with an uneven background intensity [14]. It is a mathematical gray-level morphology technique:

(1) P=I−[(I⊙S)⊕S]

where:

P: resultant image;
I: input image (transformed image);
S: structure element, disk shape, radius is 7 pixels;
⨀: morphological erosion operation; and
⨁: morphological dilation operation.

After the image subtraction operation, the peaks (MCs and noises) from the input image were easier to detect.

##### Wavelet

Discrete wavelet transformation is a popular technique for feature extraction in many areas [27]. The area of a single MC is small and usually has greater contrast than the surrounding background. Therefore, MCs have relatively high-frequency components in wavelet decomposition. This difference could provide valuable information to distinguish MCs from other tissues. To obtain the high-frequency components and the positions of MCs, we removed the low-frequency components and then reconstructed the image. To do so, we used a two-level two-dimensional (2D) wavelet transformation [19,28].

The input image was decomposed row-by-row and column-by-column using the one-dimensional wavelet transform. This step yielded four quarter-sized sub-bands, and the lowest frequency sub-band was further decomposed. The Daubechies' four-coefficient (DAUB 4) filter was more “spike-like” and needed less computation time than the other Daubechies' wavelet filters [19,28].

Two-level DAUB 4 filters were applied to the images after ILT transform. Subsequently, seven sub-bands could be obtained. We eliminated the lowest frequency sub-band on the second level and then reconstructed the filtered image. In this way, we preserved the useful information regarding MCs and removed the low-frequency components. Thus, the uneven background and low-contrast problems were alleviated by the top-hat and the wavelet filters, respectively.

##### Laws

We applied Laws filters to produce 15 Laws [29] images for further textural feature extraction [21,29]. The 15 Laws images were a composition of different low-pass, band-pass, and high-pass filters named as follows: LL, EE, SS, RR, WW, LE, LS, LR, LW, ES, ER, EW, SR, SW and RW. For instance, LL was a resultant image filtered by a low-pass filter in the x-direction and then a low-pass filter in the y-direction. These filters were created by five basic kernels as follows:

$$\begin{array}{l} \text{L}=\left[\begin{array}{lllll} 1 & 4 & 6 & 4 & 1 \end{array}\right]\\ \text{E}=\left[\begin{array}{lllll} -1 & -2 & 0 & 2 & 1 \end{array}\right]\\ \text{S}=\left[\begin{array}{lllll} -1 & 0 & 2 & 0 & -1 \end{array}\right]\\ \text{W}=\left[\begin{array}{lllll} -1 & 2 & 0 & -2 & 1 \end{array}\right]\\ \text{R}=\left[\begin{array}{lllll} 1 & -4 & 6 & -4 & 1 \end{array}\right] \end{array}$$

Therefore, LE refers to a resultant image filtered (via a convolution calculation) by a mask generated by the vector outer product L^⊗^E.

#### 2.2.3. Textural Feature Extraction (GLCM)

The gray-level co-occurrence matrix (GLCM) [30] is a well-known and popular technique for extracting texture information from images. The GLCM element *P_θ,d_(i,j)* represents the joint probability of the occurrence of gray levels *i* and *j* for a pixel pair separated by a distance d with an angle θ. There were many features listed in the literature that could be used to extract information from co-occurrence matrices. In this study, we used 14 features (with *d* = 1 and θ = 0°, 45°, 90°, and 135°) shown in Table 1 [30,31] and extracted information from a sub-image of size 16 × 16 centered on the suspected MC [14,24].

To obtain as much textural information as possible, we applied top-hat, wavelet, and Law's filters to the ILT images. Including the ILT image itself, textural information from these 18 resultant images (named as feature images) were extracted via the 14 features (each one had four angles) calculated from their corresponding GLCM. Thus, we generated a total of 56 (=14 × 4) textural features to represent a feature image.

### 2.3. Detection of MC Candidates

After image filtration (top-hat and wavelet), the candidates, including MCs and noise, could be detected. This filtration step was to limit the classification only to the candidates rather than analyze the whole breast region, which helped to reduce the computation time and decrease the false positive rate. The detection of MC candidates for top-hat- and wavelet-filtered images differed. A knowledge-based classification procedure was followed after these two filtrations.

#### Top-hat-filtered image

Our goal was to segment individual MC candidates. After top-hat filtration, the Otsu method was again used to find the threshold value on the filtered images. However, this threshold value was not used to produce a binary image but to decrease the gray level of the whole image and set any gray-values under this threshold to zero. This step was performed as follows:

(2) $$I^{\prime }\left(x,y\right)=\{\begin{array}{ll} 0 & \text{if}\;I\left(x,y\right)<T\\ I\left(x,y\right)-T & \text{otherwise} \end{array}$$

**Figure 3.**
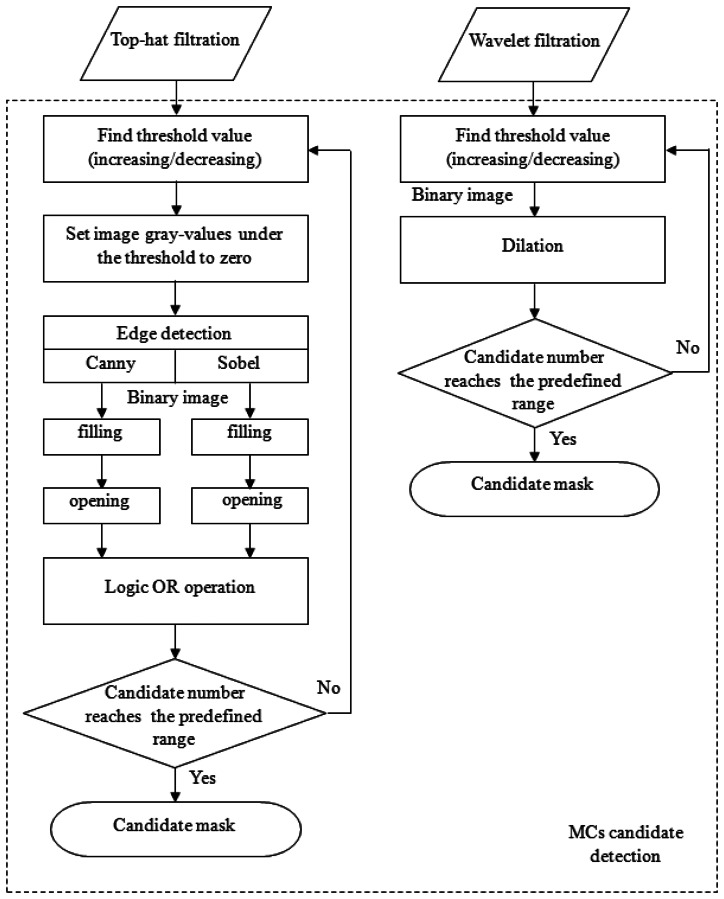
The flowchart of MC candidate detection in the top-hat- and wavelet-filtered images.

The Sobel and Canny edge detection techniques were then applied to detect the MC edges individually [14]. After edge detections, a flood-filling operation was used to fill all objects with closed contours for both results generated by the Sobel and Canny operators. Subsequently, these two filled binary images underwent a morphological open operation to break off connective candidates. Then, to reduce the number of false negatives (FNs), these two images were subjected to a logical OR operation to produce a new binary image. The reason for using two types of edge detection methods was based on the fact that we had observed that no single method could detect all of the MCs. However, this dual detection resulted in another problem: the number of candidates was significantly increased. To control the number of candidates, the above procedures were performed iteratively via either increasing or decreasing the threshold value until the number of candidates was within a predefined range. This procedure is shown in the left column in Figure 3. After this process, a binary image was obtained, which was regarded to be MC candidates.

#### Wavelet filtered image

The detection of MC candidates from the wavelet-filtered image occurred through a similar process. The reconstructed image preserved high-frequency components, which were regarded to be MCs and noise. The iterative thresholding technique described in the right column of Figure 3 was performed on the reconstructed image. The predefined range for the number of candidate were set empirically by [450, 550] and [350, 400] for top-hat- and wavelet-filtered images, respectively. This process also produced a binary image. Ultimately, these two binary images (top-hat- and wavelet-filtered images) were combined via a logical OR operation. This image was a candidate mask indicating where the candidates were located.

Figure 3 is the flowchart illustrating the process of generating a MC candidate mask using top-hat and wavelet filtering. After the candidate mask was made, a knowledge-based classification step followed to remove some FPs. Then, feature extractions were performed only on the remaining candidates so that the computation time could be considerably reduced.

### 2.4. Knowledge-Based Classification (Noise Reduction)

The output images resulting from the previous steps might contain many false positives (FPs, noise). This possibility was because the wavelet extracted high-frequency components without consideration of any shape information. The noise included breast border line sections, artificial markers, *etc*. According to their characteristics and shape information, a knowledge-based classification step was designed to remove as much noise as possible. The candidates were removed if they fit one of the following knowledge rules:

- Size: if the candidate was larger than 100 pixels. Because the MC should be small, a large calcification might not be a typical symptom of malignancy. Some artificial markers used for localization were attached to a patient's skin. These markers might cause false-positive candidates. These types of candidates were considered to be noise and were removed.
- Shape: if the shape was line-like. This determination was performed by checking the width-to-height ratio of the candidate and checking its elongation and compactness. If the elongation was larger than 3.5 and if the compactness was less than 0.38, then the candidate was considered to be noise. These numbers were set empirically. The definitions for elongation and compactness are defined as follows:
  (3) $$\text{elogation}=\text{max}\left\{\frac{\Delta x}{\Delta y},\frac{\Delta y}{\Delta x}\right\}$$
  (4) $$\text{compactness}=\frac{\text{area}}{\Delta x\times \Delta y}$$
  where Δx and Δy are the longest axes along the x- and y-direction (width and height) of the candidate, respectively.
- Single candidate in a local area: this system should be able to identify a MC cluster but not a single MC. Consequently, any isolated MCs had to be ignored. If less than three candidates were located in a 1 cm^2^ [32] area, we defined it to be an isolated MC and simply removed the hit from the candidates.

### 2.5. Automatic Optimal Feature Set Selection

There were 18 total feature images: 15 from Laws filtering, one from wavelet filtering, one from top-hat filtering, and one from the ILT transform without any filtering. Each feature image resulted in 56 features. Not all features performed well in MC detections. High-dimensional feature vectors imposed a high computational cost, as well as added a risk of over-fitting in classification [33]. Therefore, finding the most discriminatory feature set was important [34]. In this study, we used sequential forward search (SFS), sequential backward search (SBS) [35], and F-score [36] methods to select better features. SFS was a bottom-up search procedure beginning with an empty feature set and ending when all features were added; one feature at a time was added to the current feature set [14,19]. SBS was the top-down counterpart of the SFS method. It started from the complete set of features and discarded the least discriminatory feature at each stage [16,19]. The F-score selection was a simple method that measured the discrimination of two sets having real numbers [37,38]. In the F-score selection procedure, the feature with the largest F-score was included in a feature set for the following selection among the remaining available features.

The discriminatory ability of the features was determined by comparing the selection results to the ground truth using the mean square error (MSE) defined as follows:

(5) $$\text{MSE}=\frac{1}{s}\sum_{i=1}^{s}{\left(g_{i}-c_{i}\right)}^{2}$$

where *i* was the *i*th pattern to be classified, *s* was the total number of test patterns, *g_i_* was the ground truth, and *c_i_* was the classified result.

The 5-fold cross-validation from the training dataset was used to obtain an averaged MSE. A smaller MSE value meant a better feature set. Therefore, the feature subset that generated the minimum MSE was used for the training classifier.

### 2.6. SVM: Optimal Parameter Determination

SVM was capable of extracting the optimal solution with a small training set size. It was based on the principle of structural risk minimization, which aims to minimize the bound on the generalization error. Therefore, SVM tended to perform well when applied to data outside the training set [14,15,38,39]. The kernel function used here was the radial basis function (RBF). The formula for RBF is:

(6) $$K\left(x,z\right)=\text{exp}\left(-\sigma ∥x-z{∥}^{2}\right)$$

Once a kernel function had been adopted, the kernel parameters (here σ), as well as the smoothing parameter *C*, could be investigated in the cost function to obtain an optimal classifier [33].

#### Training process

To obtain an optimal parameter set, we used a grid space of (*C*, *σ*) with *log_2_ C* ϵ {−5, −3, …, 15} and *log_2_*σ ϵ 2 {−15, −13, …, 3}. For each pair (*C*, *σ*) in the search space, we conducted 5-fold cross-validation on the training set. Finally, we chose parameters with the lowest cross-validation error rate for SVM on each filtered image. All training sub-images were extracted from MC candidates. According to the ground truth, sub-images corresponding to true MCs were classed to “MC training set” (MC present), and the rest were classed to “normal training set” (MC absent). Because the “normal training set” had a larger number of images than the “MC training set,” we randomly selected training patterns from the normal training set. To control the number of FPs in a lower level which simultaneously achieving an acceptable sensitivity, the number of normal training patterns (MC absent) was five times [40] that of the number of MC training patterns (MC present). The optimal parameter set for each SVM was obtained by checking the accuracy of classification on the training dataset for each (*i*-th) MC candidate (*MC_can_*(*i*)). However, for each SVM, we had classification results (*C*_*SVM*(*j*)_(*i*) = {1 or 0}, *j* = 1,2,…,18.) on the *i*-th MC candidate, meaning that there were 18 classification results for each MC candidate, resulting in possible inconsistencies. These 18 classification results had to be combined to generate a final decision. To perform the combination, we defined a decision rule as follows:

(7) $$MC_{\text{can}}(i)=\{\begin{array}{ll} \text{True}, & \text{if}\sum_{j=1}^{18}C_{\text{SVM}(j)}(i)\geq N\\ \text{False}, & \text{otherwise} \end{array},$$

where ‘true’ denoted that the *i*-th MC candidate was a true MC and *N* was a threshold. We found if N = 1, there were many FPs, and if N = 18, the sensitivity was too low. To study the relationship between N and sensitivity, a fROC curve was used, and its results are shown in Section 3.

#### Test process

A total of 18 SVMs were trained, and their corresponding optimal parameter sets were obtained as described in the training process. The test dataset was pre-processed using the same procedures as in the training process, including ILT, wavelet, top-hat, and Laws filtering. Then, binary images containing MC candidates were obtained. The knowledge-based classification was followed to remove false positives. The remaining candidates were subjected to the textural feature extractions using the optimal feature selection method for each corresponding feature image found in the training process (marked gray in Table 2). These 18 feature images (extracted from 18 filtering images centered in each candidate) were classified by their corresponding SVMs, and each SVM gave a classification result for every individual candidate. The fROC curve was used to study the system accuracy and the false positive rate.

### 2.7. Performance Evaluation

Regarding the performance evaluation of MCCs, we applied the evaluation criteria proposed by [32]. Their two criteria are listed as follows.

1. The ratio of the overlapping area made by the automatic detection and the ground truth made by professional physicians is greater than 50%, and the detected area is not larger than four times of the ground truth area.
2. At least three MCs are detected within 1 cm^2^ of the nearest neighbor to form a MC cluster.

According to these criteria, we calculated the system sensitivity with respect to FPs per image to evaluate the performance of the proposed system.

## 3. Results

**Figure 4.**
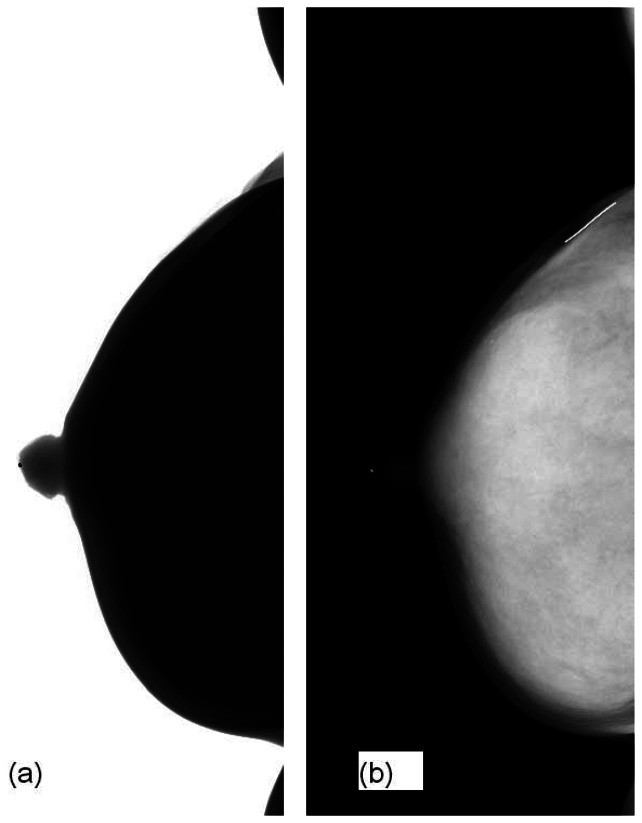
The image before and after ILT. (**a**) The raw image. (Image depth: 14-bit); (**b**) The ILT image (image depth: 12-bit).

Figure 4 shows our image before and after ILT. The raw image is not for clinical use. ILT could not only reduce the image depth from 14-bit to 12-bit but also did performed image enhancement in the breast region so that MCs could be viewed visibly.

**Figure 5.**
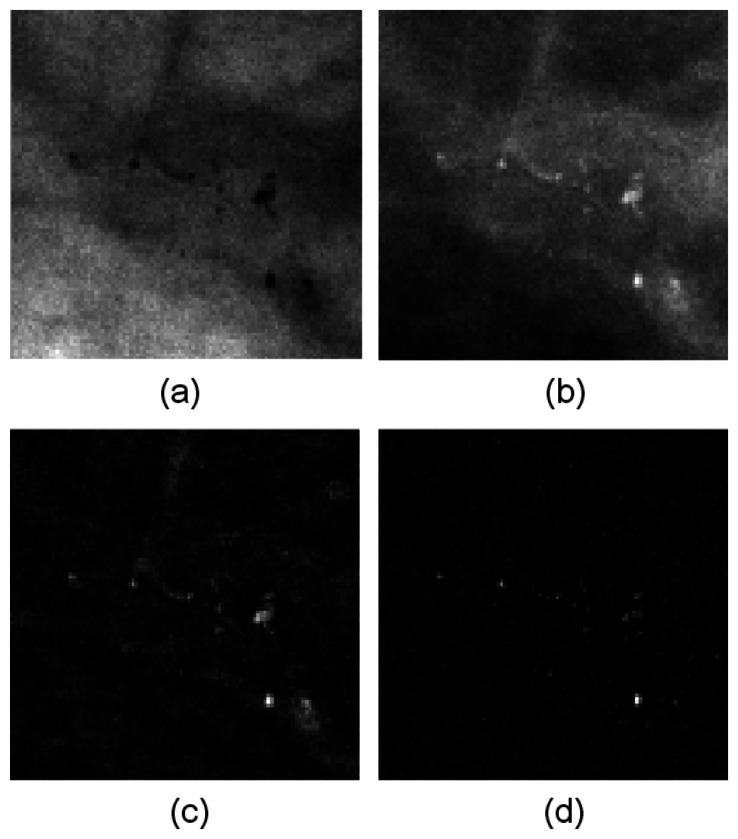
The image before and after transformation and filtering. (**a**) The raw image; (**b**) The ILT image; (**c**) After top-hat filtering; (**d**) After wavelet filtering.

Figure 5 presents the image before and after filtering. Figure 5(a) is the ILT image without any filtering. Figure 5(b,c) are the filtering results from the top-hat and wavelet methods, respectively. The MCs were enhanced noticeably enhance in the resulting images, and other homogeneous regions were ignored.

**Figure 6.**
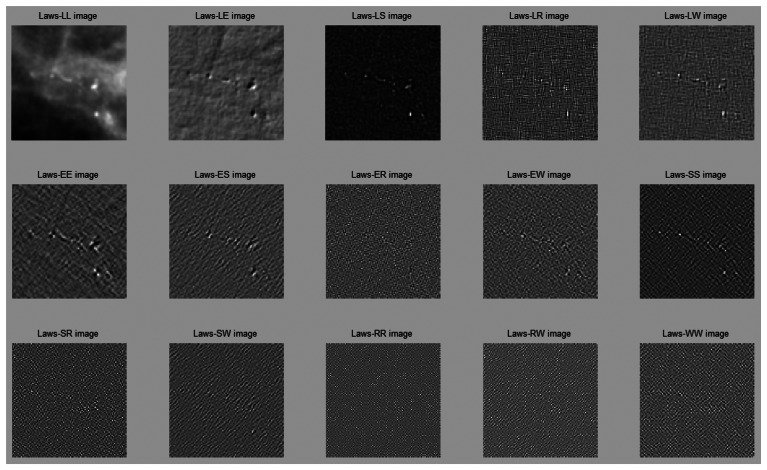
The results after Laws filtering.

Figure 6 illustrates the image results after Laws filtering.

Twenty-six cases contained more than 50 images in the training dataset were used for studying the performance of feature selection. The performance results of 18 different filtered images with respect to three different feature selection methods are shown in Table 2. To understand the effect of feature selection, we also used all features (56 features: listed in the fourth row in Table 2) to compare the three selection methods. The performance was evaluated by Az (area under curve) values. In Table 2, the A_z_ values marked in gray represent the best results for each column. From Table 2, we noted that the texture features extracted from a LS image with SBS feature selection (in which 22 features were selected) achieved the best performance. The LE image with SFS (in which 14 features were selected) was the second best. The third best was the top-hat filtered image (in which all features were selected). Notably, there were only three out of eighteen cases for which the system achieved the best performance using all the features. In most situations, the performance was improved when feature selection was used. Our results have confirmed that a hyper-feature dimension risked over-fitting problems in classification [33]. Furthermore, the SFS and SBS methods appeared to be more effective than the F-score method. The optimal SVM parameters chosen for the 18 feature images are shown in Table 3. These parameters were obtained using the training dataset. The results demonstrate that different feature images had different optimal parameter sets for SVM. The optimal parameter sets were applied in the test dataset.

**Figure 7.**
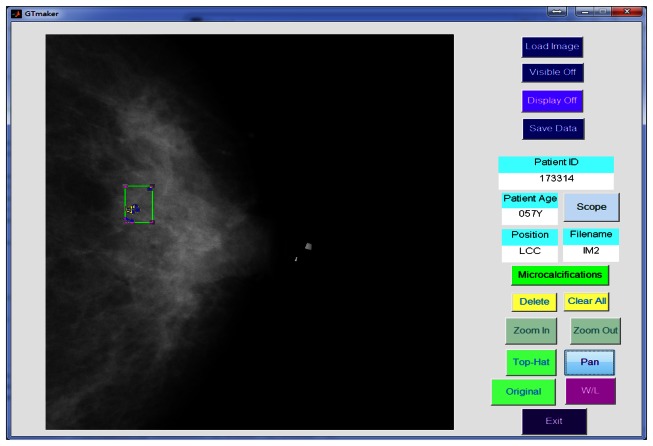
A GUI was designed for manual identification of MCs and MCCs.

In this study, a GUI (graphic user interface) was designed in the Matlab platform [41] to generate a gold standard. The manual identification of the MCs was performed by two independent radiologists. Using the GUI, the expert could identify individual points of MCs in the mammogram and drew a rectangle area to define a MC cluster manually, as shown in Figure 7. Another GUI program was developed for the process described in this paper to detect the MCs and further marked the clustered MCs fully automatically.

**Figure 8.**
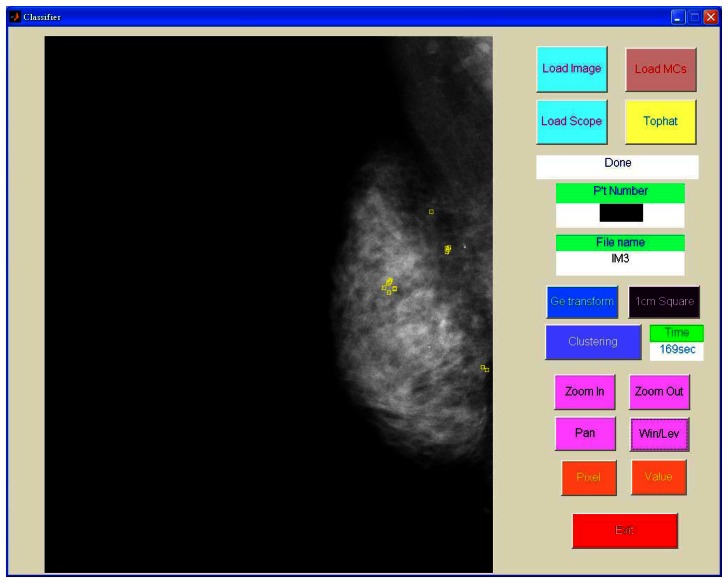
The automated MC detection results.

**Figure 9.**
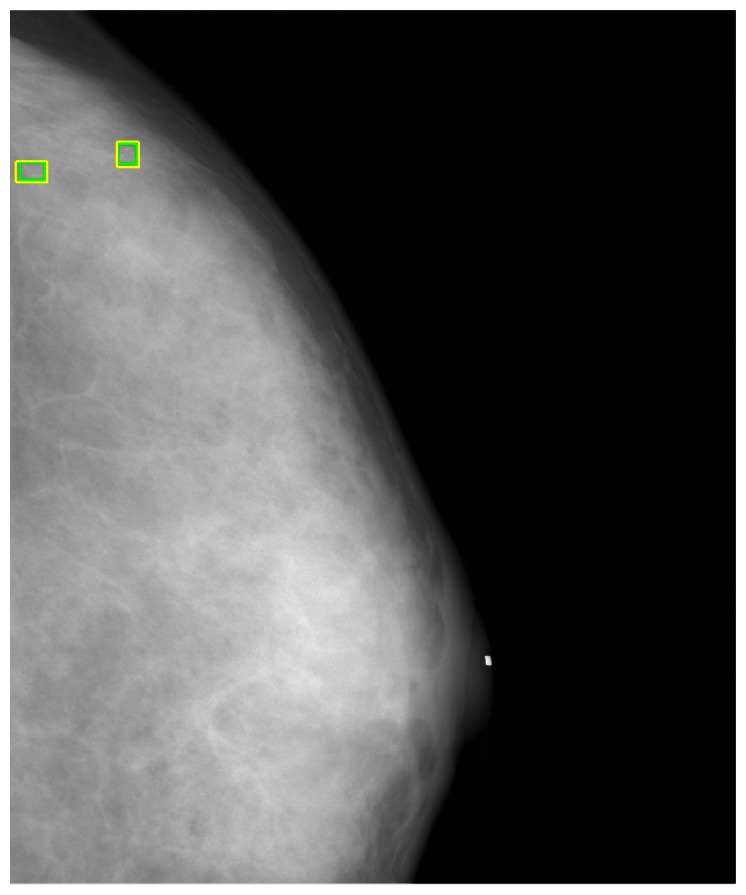
The automated MC cluster detection results.

Figure 8 shows the detected individual MCs marked by small squares. Figure 9 shows the detected MC cluster marked by rectangles. There are four rectangles in Figure 9, two of which were made manually and another two marked automatically. The automated results were very similar to the manually selected results. These two MCC were successfully detected and marked.

**Figure 10.**
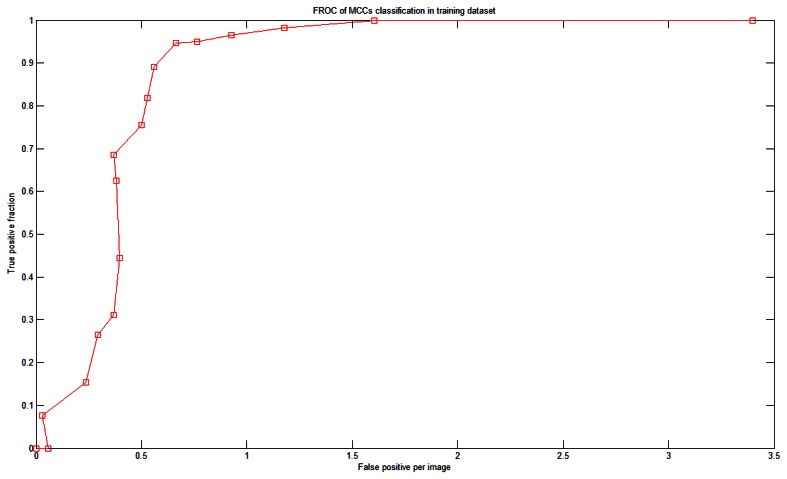
The fROC curve for the training dataset.

To study the relationship between N and sensitivity (Section 2.6), a fROC curve was used. Figure 10 shows the relationship between N and the sensitivity (and FPs) of the training dataset. There were 18 SVMs. If N = 1 then the system achieved 100% sensitivity; however, the FP rate was 3.4 per image. When N was increased, the sensitivity decreased and so did the number of FPs. Figure 10 is the fROC curve. This curve was generated by testing integer N in the range of [1,18]. We defined the following criterion to determine which N to use: the largest sensitivity whose FP is less than 1. In Figure 10, the optimal value was N = 4.

**Figure 11.**
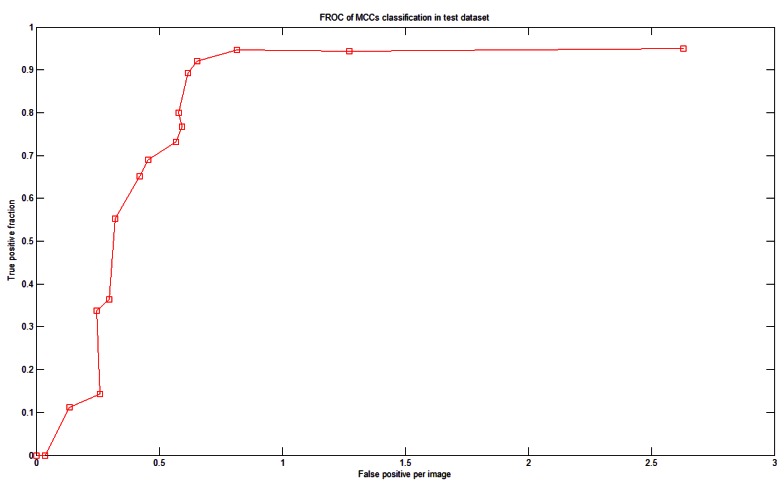
The fROC curve for the test dataset.

There were more than 60 MCCs from more than 50 mammograms in the test dataset. To test the system performance we generated a fROC curve of the detection results using the test dataset (Figure 11). Based on the optimal parameters and settings determined in the training dataset, our system achieved 92% sensitivity with 0.65 FPs per image in the test dataset.

## 4. Discussion

We found that in our dataset some MC clusters were very small, with sometimes only three individual MCs in a cluster. Therefore, when one of the three MCs was misclassified by the proposed system, the cluster was not selected according to Kallergi's criteria. This problem might most likely be one reason that the sensitivity was relatively lower than in [15]. However, our method still maintained an acceptable detection performance and low FP rate. Table 4 illustrates a comparison between different methods. According to Table 4, the proposed method outperformed the existing methods.

**Figure 12.**
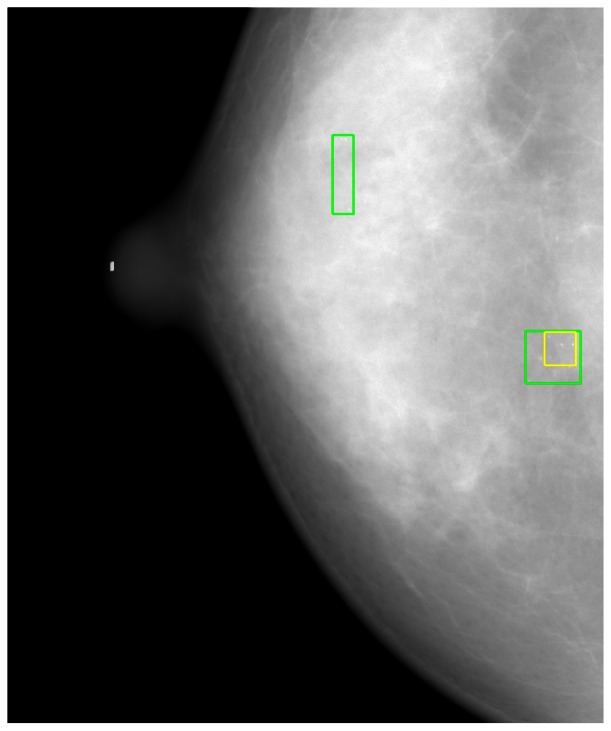
Examples of a false positive (lower rectangle) and a false negative (upper rectangle).

Another reason for FPs was the strict conditions of Kallergi's criteria. Based on Kallergi's criteria, if the automated marked area (MCC) was less than 50% of the manually marked area, then it was counted as a FP, and the FN number was simultaneously increased by one because the MCC was not detected (see Figure 12). In Figure 12, two lower rectangles are drawn on the image. The smaller one was generated using automated MCC detection, and the larger one indicates the manually marked MCC. Because the automatically marked area was less than 50% of the manually marked area, it was counted as a FP even though MCC was correctly detected. Simultaneously, the FN was increased by one, which further reduced the sensitivity. However, we also identified a MCC that was not found (a FN), as denoted in Figure 12 by the upper rectangle.

El-Naqa [15] had 50,000 MC-absent training patterns. However, we used only five times the number of MC-containing training patterns (*i.e.*, approximately 5,300 MC-absent patterns). Our system achieved good performance with lower FP. If we allowed the FP to be 0.8 MCC per image, then our system could achieve a sensitivity of 94.7%, as shown in Figure 11. The proposed system has made many efforts in optimization such as determining the optimal parameter sets of the SVMs and the optimal features in the corresponding feature images. Both processes were fully automated, which could be a reason that our system demonstrated better performance than the previous methods listed in Table 4.

## 5. Conclusions

This study describes the development of a CADe system to detect MC clusters for early breast cancer detection. The textural features extracted from wavelet- and Laws-filtered images were useful in discriminating MCs from other normal tissues or noise. The SVM with the optimal parameter settings and automated feature selection method performed well for MC and MCC detection. The proposed system achieved an *Az* 0.9897 for MC detection and 92% sensitivity, with 0.65 FPs per image for MCCs. In the future, we will use mammograms from different modalities to evaluate the stability of our system. This system is helpful for clinical physicians in the routine work of mammogram screening to reduce their reading load.

## Figures and Tables

**Table 1.** Fourteen textural features were extracted from GLCM [30].

1	Contrast	6	Prominence	11	Sum average
2	Homogeneity	7	Inverse difference moment	12	Sum entropy
3	Energy	8	Entropy	13	Difference entropy
4	Correlation	9	Intensity	14	Difference variance
5	Shade	10	Sum of squares variance		

**Table 2.** Performances of different selection methods with respect to 18 feature images in the training process.

	**ILT**	**Top-hat**	**Wavelet**	**LL**	**EE**	**SS**	**RR**	**WW**	**LE**
SFS	0.9758	0.9749	0.9719	0.9755	0.9686	0.9419	0.6749	0.8459	0.9820
SBS	0.9745	0.9764	0.9729	0.9756	0.9707	0.9503	0.6866	0.8531	0.9816
F-score	0.9737	0.9774	0.9726	0.9753	0.9730	0.9339	0.6758	0.8588	0.9751
ALL*	0.9728	0.9775	0.9702	0.9748	0.9758	0.9295	0.6812	0.8403	0.9732
	LS	LR	LW	ES	ER	EW	SR	SW	RW

SFS	0.9882	0.9119	0.9646	0.9267	0.9014	0.9559	0.8422	0.9030	0.7529
SBS	0.9896	0.9199	0.9619	0.9499	0.9010	0.9435	0.8465	0.9057	0.7408
F-score	0.9874	0.8994	0.9634	0.9503	0.9019	0.9275	0.8339	0.9025	0.7408
ALL	0.9878	0.9006	0.9570	0.9271	0.9022	0.9275	0.8339	0.9000	0.7408

* ALL indicates that all 56 features were used in SVM; The numbers in the table represent the area under curve (Az) of ROC.

**Table 3.** The optimal parameter sets of SVM with respect to the 18 feature images.

**Parameters**	**ILT**	**Top-hat**	**Wavelet**	**LL**	**EE**	**SS**	**RR**	**WW**	**LE**
*log*_2_*C*	13	9	2	14	5	12	14	13	15
*log*_2_σ	−12	−10	−7	−12	−10	−12	−12	−12	−13
**Parameters**	**LS**	**LR**	**LW**	**ES**	**ER**	**EW**	**SR**	**SW**	**RW**

*log*_2_*C*	3	6	2	13	8	7	8	14	11
*log*_2_σ	−7	−8	−6	−12	−12	−10	−10	−14	−11

**Table 4.** Comparison between different methods.

	**No. of Mammograms**	**No. of Clusters**	**No. of Cases**	**Sensitivity**	**Area Under the Curve**	**False Positive**
Our method	111	135	52	92%	0.99 (MC)	0.65 per image
Baum (*) 2002 [42]	187	N/A	63	87.3%	N/A	0.61 per image
El-Naqa 2002 [15]	76	N/A	N/A	94%	N/A	1 per image
Cheng 2004 [17]	40	105	21	90.5% (95/105)	N/A	1 per image
Wei 2009 [16]	200	N/A	104	N/A	0.82	N/A

(*) Commercial product: Image Checker V2.3, R2 Technology, Los Altos, Calif.
